# Capturing the dynamic nascent transcriptome during acute cellular responses: The serum response

**DOI:** 10.1242/bio.019323

**Published:** 2016-05-26

**Authors:** Killeen S. Kirkconnell, Michelle T. Paulsen, Brian Magnuson, Karan Bedi, Mats Ljungman

**Affiliations:** 1 Department of Radiation Oncology, University of Michigan Comprehensive Cancer Center, andTranslational Oncology Program, University of Michigan, Ann Arbor, MI 48109, USA; 2 Department of Human Genetics, University of Michigan Medical School, Ann Arbor, MI 48109, USA; 3 Department of Environmental Health Sciences, School of Public Health, University of Michigan, Ann Arbor, MI 48109, USA

**Keywords:** Regulation of transcription, Transcription elongation, Signal transduction, Gene length

## Abstract

Dynamic regulation of gene expression via signal transduction pathways is of fundamental importance during many biological processes such as cell state transitioning, cell cycle progression and stress responses. In this study we used serum stimulation as a cell response paradigm to apply the nascent RNA Bru-seq technique in order to capture early dynamic changes in the nascent transcriptome. Our data provides an unprecedented view of the dynamics of genome-wide transcription during the first two hours of serum stimulation in human fibroblasts. While some genes showed sustained induction or repression, other genes showed transient or delayed responses. Surprisingly, the dynamic patterns of induction and suppression of response genes showed a high degree of similarity, suggesting that these opposite outcomes are triggered by a common set of signals. As expected, early response genes such as those encoding components of the AP-1 transcription factor and those involved in the circadian clock were immediately but transiently induced. Surprisingly, transcription of important DNA damage response genes and histone genes were rapidly repressed. We also show that RNA polymerase II accelerates as it transcribes large genes and this was independent of whether the gene was induced or not. These results provide a unique genome-wide depiction of dynamic patterns of transcription of serum response genes and demonstrate the utility of Bru-seq to comprehensively capture rapid and dynamic changes of the nascent transcriptome.

## Abstract (precis)

**Summary:** This study used nascent RNA Bru-seq to provide an unprecedented comprehensive view of the dynamic transcriptome as cells reset homeostasis to adjust to new environmental conditions.

## INTRODUCTION

Signal transduction cascades are critical regulators of cell processes such as differentiation, proliferation, and stress responses. These cascades activate preexisting transcription factors, which in turn induce various groups of targets genes. Some of these target genes are also transcription factors that, after the completion of transcription and translation, go on to regulate their own sets of target genes. These series of regulatory and transcriptional networks allow for an initial triggering event to produce complex global gene expression changes in a temporal and dynamic way.

Growth factor activation is a widely used system for studying rapid changes in gene expression. Fibroblasts grown in serum-free media enter a G_0_/G_1_ state, and subsequent addition of serum to these cells results in global gene expression changes as cells prepare to progress through the cell cycle again (Winkles, 1997). Studies analyzing the genome-wide early transcriptional response to serum stimulation indicate that response genes exhibit various temporal expression patterns (Amit et al., 2007; Iyer et al., 1999; Tullai et al., 2007). In addition to recruitment and initiation of transcription by RNA Polymerase II (RNAPII), several additional mechanisms are thought to influence gene expression timing, including release of paused polymerases, elongation progression, termination, and polyadenylation (Jonkers and Lis, 2015). These various points of regulatory control, which differ for individual response genes, make it challenging to unravel the complex global patterns of transcriptional activation and repression.

Methods such as microarrays and RNA-seq are excellent for detecting polyadenylated mRNA or changes in the total mRNA pool, however, the detection of these changes may not directly reflect the initial transcriptional response. Therefore we used Bru-seq, a nascent RNA sequencing technique (Paulsen et al., 2014, 2013), to focus on changes in transcriptional initiation genome-wide following serum stimulation of human fibroblasts. By measuring changes in the nascent RNA production emanating from transcription start sites (TSSs), we could identify genome-wide dynamic changes in productive transcription initiation. Our data indicate that there are several distinct gene expression patterns of induction and repression that occur in response to serum stimulation, and genes in distinct cellular pathways often exhibit similar response patterns. Furthermore, by measuring the movement of induced or repressed transcription waves through the bodies of large genes we found that the transcription machinery accelerates as it traverses genes. This study was able to explore the global dynamics of RNA production in unparalleled detail during the early serum response and showcases the unique utility of Bru-seq in capturing genome-wide transcriptional dynamics.

## RESULTS

### Immediate and sustained effects of serum stimulation on transcription initiation

**Fig. 1.**
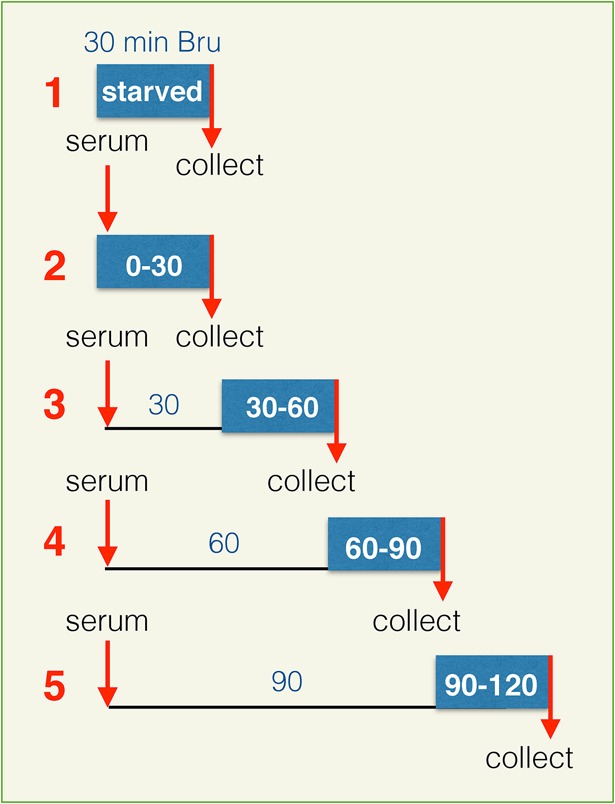
**Experimental outline.** Bromouridine labeling (2 mM) of nascent RNA was performed for 30 min (blue field) on (1) starved human fibroblasts, or (2-5) at different times after serum addition. Cells were maintained in serum-free media for 48 h prior to treatments.

In this study, we used Bru-seq to explore the dynamic landscape of the nascent transcriptome as human fibroblasts responded to serum stimulation following a 48 h period in serum-starved conditions (Fig. 1). Cells were incubated with 2 mM bromouridine (Bru) for 30 min to label nascent RNA either in serum-starved cells, or during 30 min intervals following serum addition. At the end of the labeling periods, cells were lysed in Trizol and total RNA was isolated. The Bru-labeled nascent RNA was captured from the total RNA using anti-BrdU antibodies conjugated to magnetic beads, and then converted into cDNA libraries and deep sequenced. Exploring nascent RNA production using Bru-seq allowed us to capture the dynamic landscape of transcription alterations during the first 2 h of the serum response. By mapping the reads for each 30 min labeling period, we were able to visualize active transcription across individual genes. To focus on changes in productive initiation, we measured the abundance of reads within the whole gene for those 30 kb or shorter, and within the first 30 kb for genes longer than 30 kb. We chose a 30 kb window because we expected that based on an average elongation rate of 1.4 kb/min (Veloso et al., 2014), RNAPII would have fully traversed the sampling area during the 30 min labeling period. Importantly, this approach made it possible to assess the initiation rate for very large genes for which rate otherwise may have been severely underestimated if transcription within the full length of the gene was considered.

**Fig. 2.**
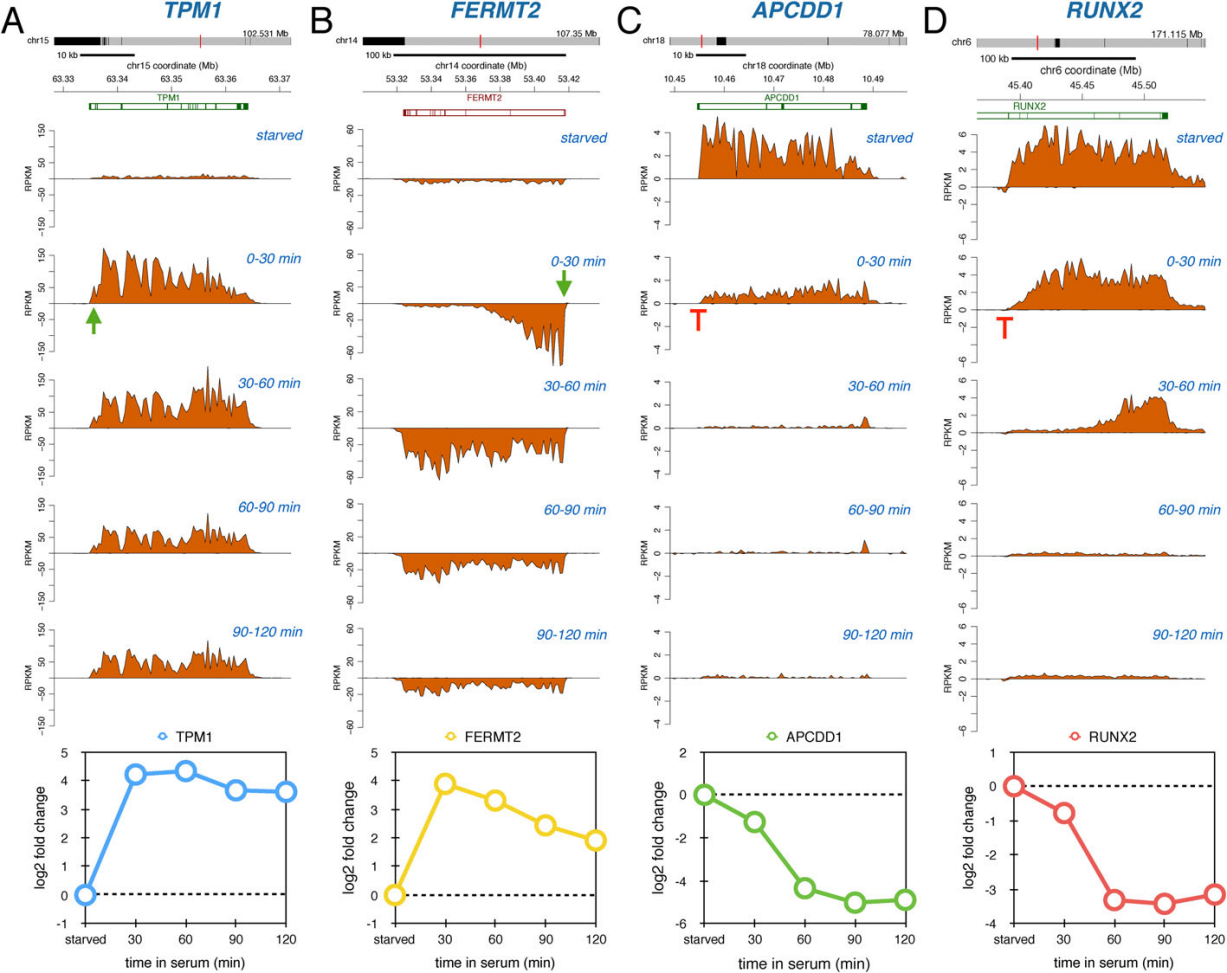
**Immediate serum-response genes.** Bru-seq traces for *TPM1* (A), *FERMT2* (B), *APCDD1* (C) and *RUNX2* (D) during starved conditions and after different periods of serum stimulation. Annotated genes are shown at the top in either red or green. The positive *y*-axis represents plus-strand signal of transcription moving from left to right and the negative *y*-axis represents minus-strand signal of transcription moving from right to left. Transcription induction is indicated by a green arrow and transcription repression is indicated by a red T. The graphs at the bottom depict the log2-fold change values calculated within the first 30 kb of the genes for each labeling period.

We observed that some genes, such as *TPM1* encoding an actin skeleton protein, were immediately upregulated following serum addition, visible through an increase in transcription reads across the entire gene (Fig. 2A). This increase was observed during each labeling period. The *FERMT2* gene encoding a scaffolding protein behaves in a very similar manner, but because this gene is considerably longer we only observe transcription reads at the 5′ end of the gene during the first labeling period (Fig. 2B); however, during the subsequent labeling period (30-60 min), the transcription wave had reached the 3′ end of the gene. One advantage of nascent RNA Bru-seq over traditional RNA-seq, which measures steady-state RNA, is that it can capture rapid inhibition of transcription very efficiently since it does not rely on the degradation of pre-existing RNA for detection of inhibition. For example, the *APCDD1* gene coding for a protein that inhibits WNT signaling was actively transcribed during serum starvation but was rapidly repressed upon serum addition (Fig. 2C). *RUNX2*, encoding a transcription factor, was also repressed by serum and due to its considerable length (>100 kb) transcription at the end of the gene was unaffected until 60-90 min after serum addition (Fig. 2D). Thus, even though genes can be induced or repressed immediately after serum addition, there is a time delay before the generation or loss of full-length products that is proportional to their gene lengths.

### Transient effects of serum stimulation on transcription initiation

**Fig. 3.**
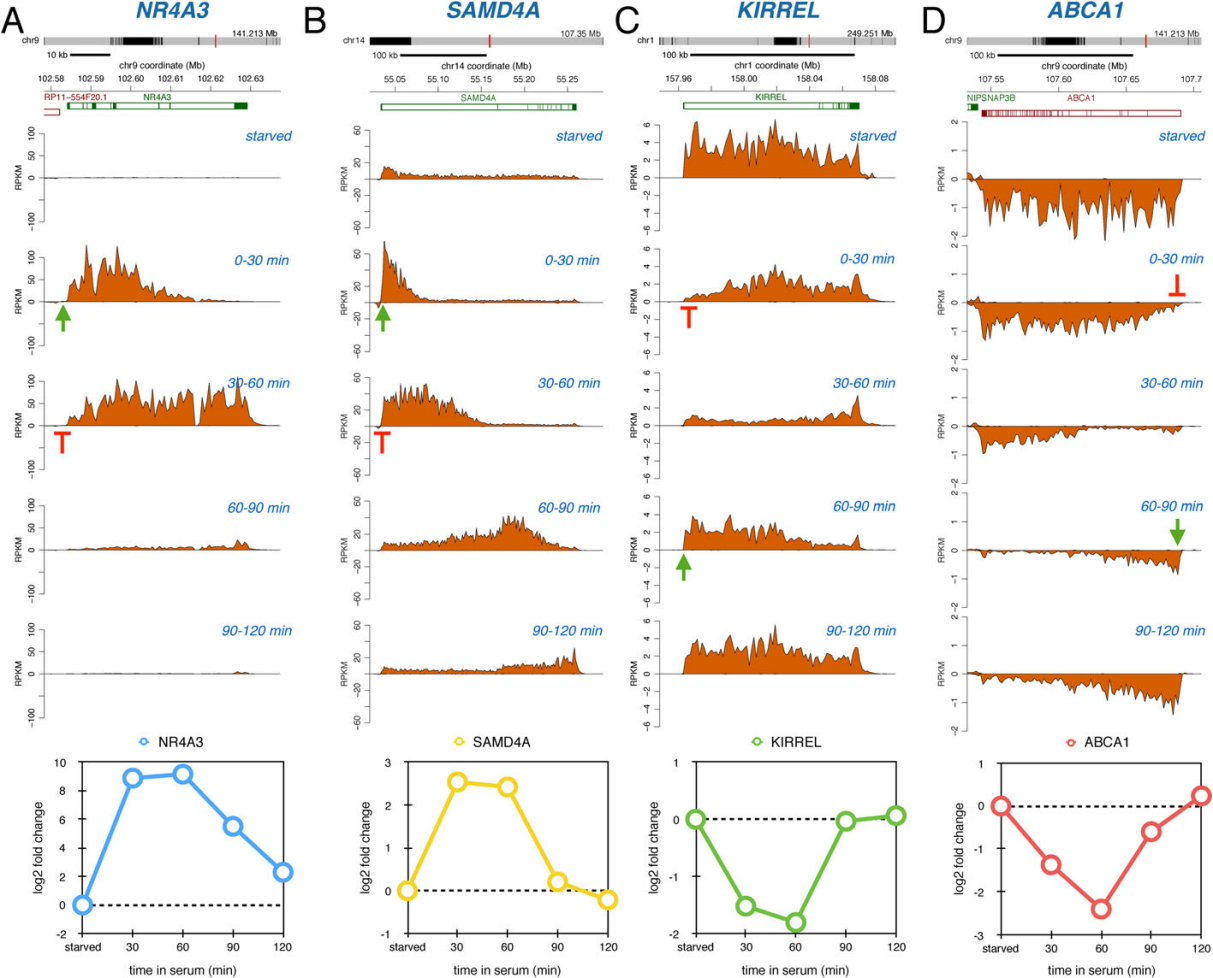
**Transient responding genes following serum stimulation.** Bru-seq traces for *NR4A3* (A), *SAMD4* (B), *KIRREL* (C) and *ABCA1* (D) during starved conditions and during different periods following serum addition. Data representation as in Fig. 2.

While the previously described genes displayed sustained transcriptional induction or repression following serum stimulation, other genes demonstrated transient regulation. For example, the transcription factor gene *NR4A3* was immediately induced after serum addition, but transcription returned to serum-starved levels within 60 min after stimulation (Fig. 3A). *SAMD4A*, encoding a RNA-binding protein, was also transiently induced and transcription returned to serum-starved levels after 60-90 min. As the *SAMD4A* gene is very long (>200 kb), there was a delay before the 3′ end of the gene experienced the effects of this brief pulse of transcriptional induction (Fig. 3B). We also observed genes such as *KIRREL,* encoding a signaling protein (Fig. 3C), and *ABCA1,* encoding a protein involved in cholesterol transport (Fig. 3D), that showed inhibition of productive initiation after serum addition followed by a reset to baseline expression.

### Delayed effects of serum stimulation on transcription initiation

**Fig. 4.**
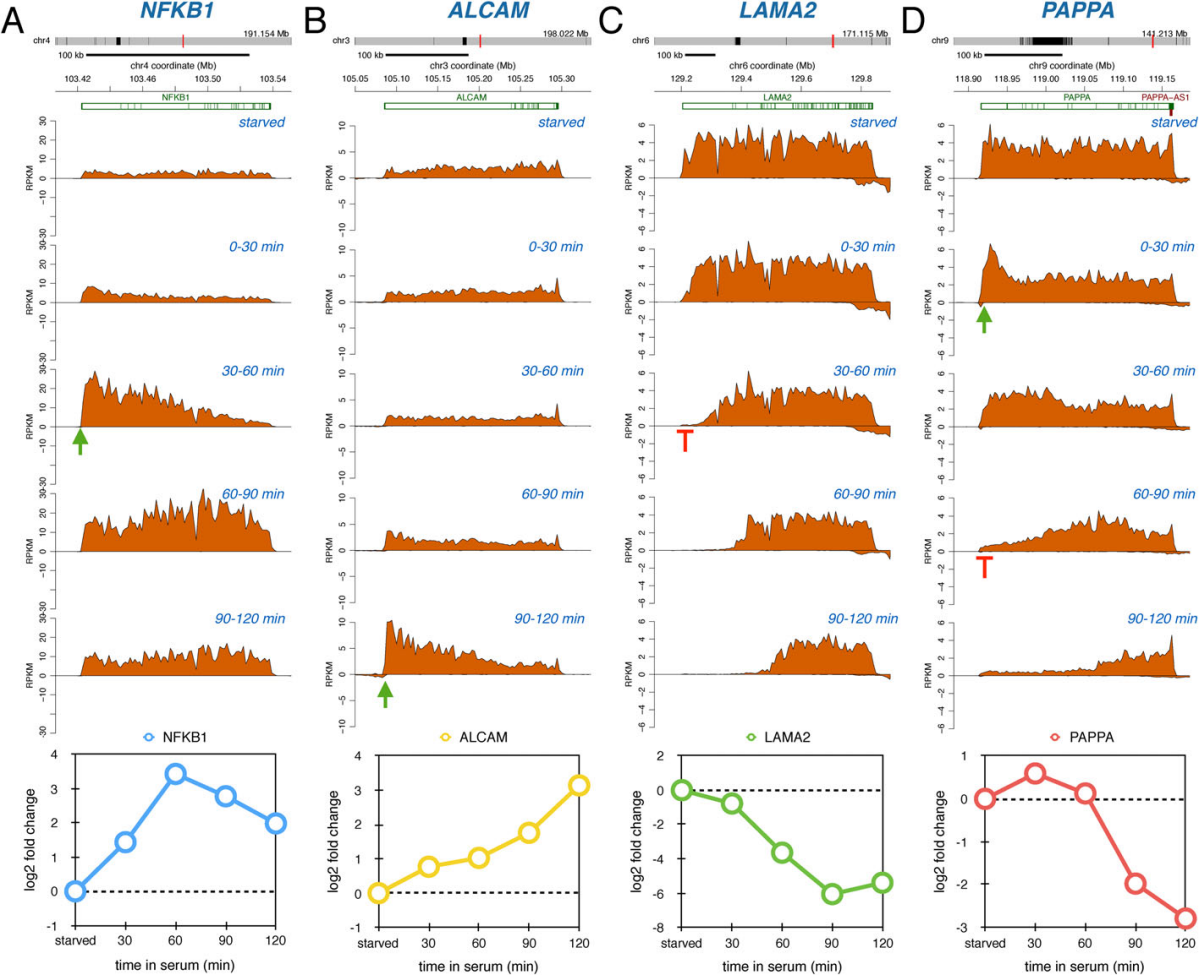
**Delayed serum-response genes.** Nascent RNA sequencing reads for *NFKB1* (A), *ALCAM* (B), *LAMA2* (C) and *PAPPA* (D) during starved conditions and during different periods following serum addition. Data representation as in Fig. 2.

Certain genes showed a delayed transcriptional response following serum activation. For example, transcriptional induction of the transcription factor gene *NFKB1* peaked during the 30-60 min labeling period (Fig. 4A) and induction of *ALCAM*, encoding a cell adhesion protein, peaked during the 90-120 min labeling period (Fig. 4B). The *LAMA2* gene, encoding a basement membrane protein, was repressed 30-60 min after serum addition (Fig. 4C), and the metalloproteinase encoding gene *PAPPA* showed a brief modest initial induction followed by repression 60-90 min after serum addition (Fig. 4D). These delayed responses may be regulated by transcription activators or repressors that are transcriptionally induced during the immediate response to serum.

### Genome-wide patterns of transcription regulation following serum stimulation

**Fig. 5.**
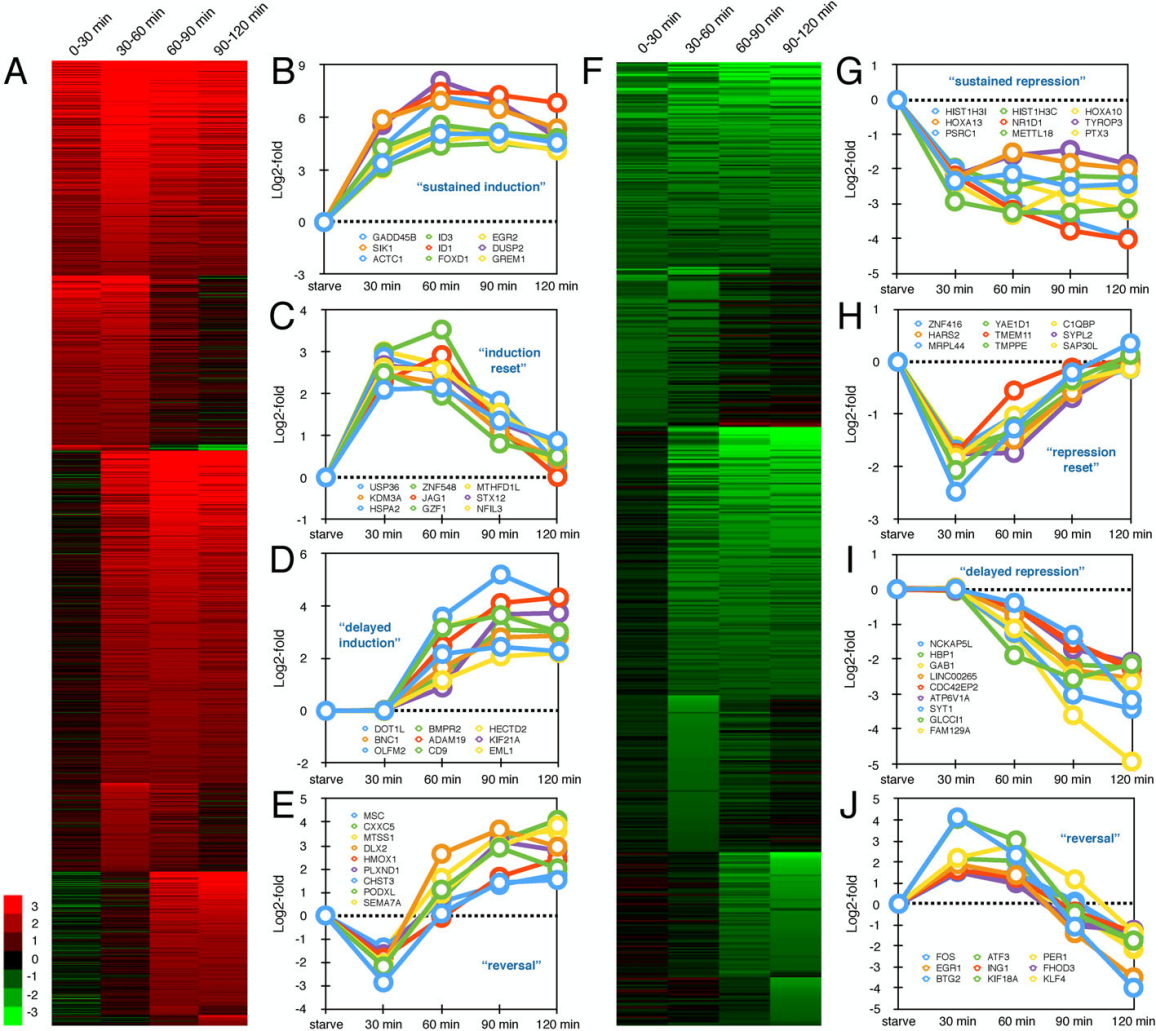
**Dynamics of the nascent transcriptome following serum stimulation.** Heat maps of induced (A) and repressed (F) genes in response to serum stimulation based on log2-fold change values within the first 30 kb of genes. Examples of gene groupings exhibiting various transcriptional patterns are shown in graphs (B-E) and (G-J).

After observing this variety of transcriptional responses, we went on to perform a genome-wide analysis of serum-induced transcriptional changes. Using a two-fold change cutoff for serum-induced transcriptional change in the first 30 kb of expressed genes, we observed 1417 genes upregulated (Fig. 5A) and 636 genes downregulated (Fig. 5F) in at least one labeling period following serum stimulation. Heat maps were generated for these serum response genes based on the fold-change values from each serum-stimulated labeling period compared to starved cells. We classified serum response genes based on transcription patterns near the TSS during the first 2 h following serum stimulation using the following categories: sustained induction or repression (Fig. 5B,G), induction or repression followed by a return to baseline expression (Fig. 5C,H), delayed induction or repression (Fig. 5D,I), and both induction and repression (Fig. 5E,J). Enrichment of genes involved in nucleosome assembly was linked to a broad sustained repression of the large histone gene cluster on chromosome 6 by serum (Fig. S1). These results, which could only be obtained by a nascent RNA sequencing approach such as Bru-seq, show the intricate patterns by which cells regulate transcription in response to a major cell stimulus such as serum. It also highlights the importance of measuring transcription in a continuous manner following exposure to the stimulus in order to capture all transient transcription events. A novel finding of this study was that the patterns of transcription repression mirrored the patterns of transcription induction suggesting that these opposite outcomes are regulated by similar mechanisms.

### Functional analysis of the serum response

**Fig. 6.**
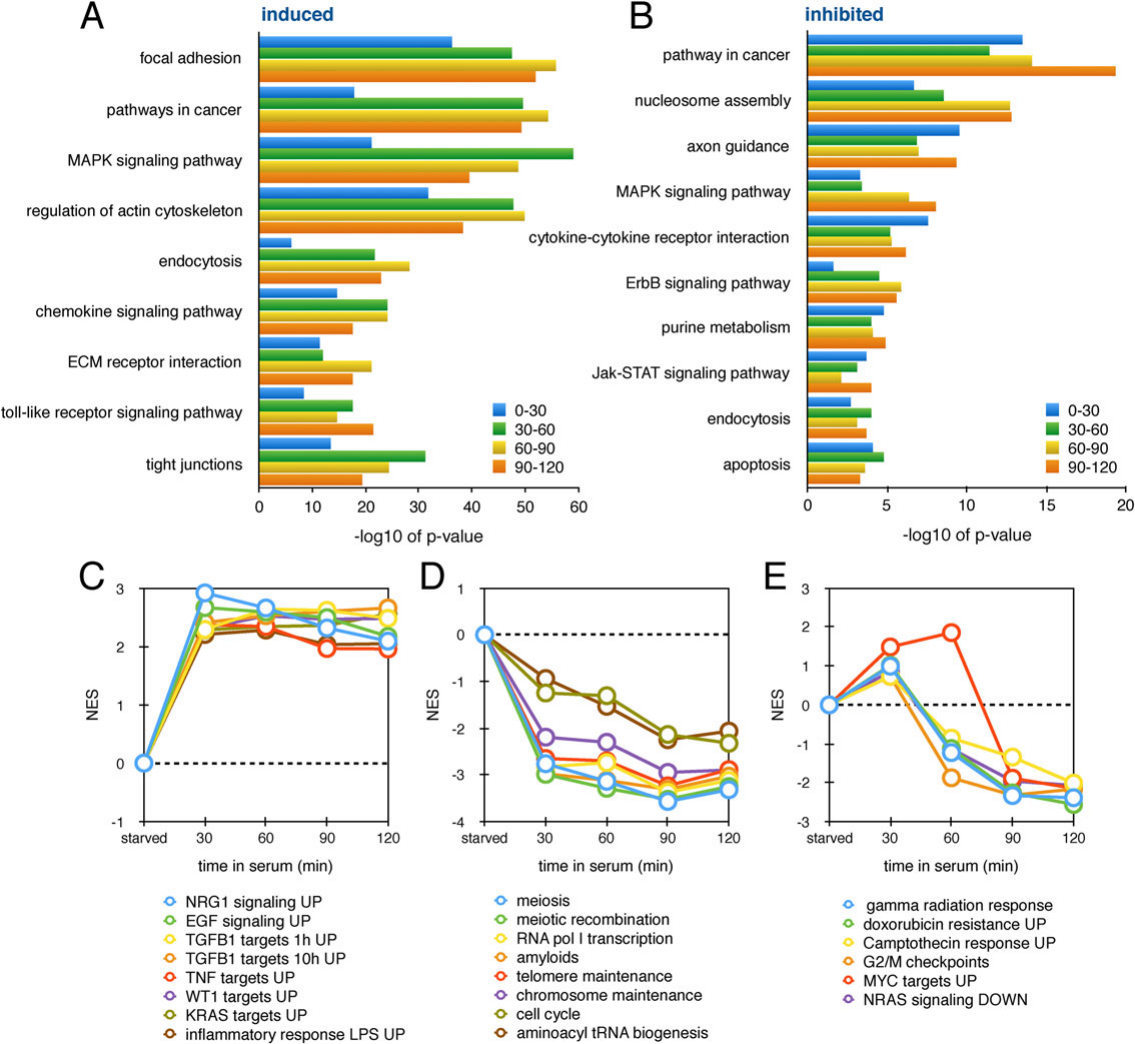
**Gene set enrichment analysis of serum response genes.** Enriched pathways identified by DAVID in induced (A) and inhibited (B) gene sets during each serum-stimulated labeling period. The −log10 values of *P*-values are displayed. Enriched pathways identified by GSEA in induced (C), inhibited (D), and induced then inhibited (E) gene sets during each time point. Normalized enrichment scores (NES) are displayed.

We utilized the database for annotation, visualization and integrated discovery (DAVID) functional annotation tool to explore whether the serum response genes identified by Bru-seq were enriched for certain functional pathways, including known pathways related to serum activation. First we focused on the group of transcriptionally induced genes to identify potential pathways induced in response to serum stimulation. We found significant enrichment of genes related to cellular structures such as ‘focal adhesion’, ‘tight junctions’, ‘actin cytoskeleton’, and ‘extracellular matrix’ (Fig. 6A; Fig. S2), which are structures known to be important during the serum response and wound healing (Cooper et al., 2004; Iyer et al., 1999). Genes involved in various signaling pathways such as MAPK, chemokine, toll-like receptor, and TGF-β signaling were also enriched following serum stimulation. Enrichment of these pathways was seen during each labeling period.

Next we examined our set of transcriptionally repressed genes to identify potential pathways which were downregulated in response to serum activation. We observed enrichment of genes involved in signaling pathways including MAPK, ErbB, Jak-STAT, and cytokine receptor signaling (Fig. 6B; Fig. S2). While the genes involved in these pathways were enriched during each labeling period, genes in other pathways had higher enrichment scores only during the later labeling periods. These pathways included ‘nucleotide excision repair’, ‘p53 signaling’, ‘cell cycle’, ‘WNT signaling’, and ‘aminoacyl tRNA biosynthesis’, (Fig. S3).

Additionally, we performed a gene set enrichment analysis (GSEA) for each labeling period (Subramanian et al., 2005). Expressed genes during a given period were ranked according to log2-fold changes in expression compared to starved cells, and this ranked gene list was used to calculate normalized enrichment scores (NESs) for gene sets. Gene sets with high NESs during each labeling period, indicating sustained increased transcription levels, included genes induced by neuregulin (NRG1), estrogen growth factor (EGF), transforming growth factor beta (TGF-β), tumor necrosis factor (TNF), Wilms tumor 1 (WT1), KRAS, and the inflammatory response (Fig. 6C). Gene sets with low NESs during each labeling period, indicating sustained decreased transcription levels, included those related to meiosis, telomere and chromosome maintenance, RNA pol I transcription, and amyloids (Fig. 6D). Genes involved in the response to ionizing radiation, resistance to the chemotherapeutic drugs doxorubicin and camptothecin, the G2/M checkpoint, as well as genes induced by MYC, and genes repressed by NRAS were initially induced in response to serum but at later time points were repressed (Fig. 6E).

### DNA damage repair and signaling genes inhibited by serum stimulation

**Fig. 7.**
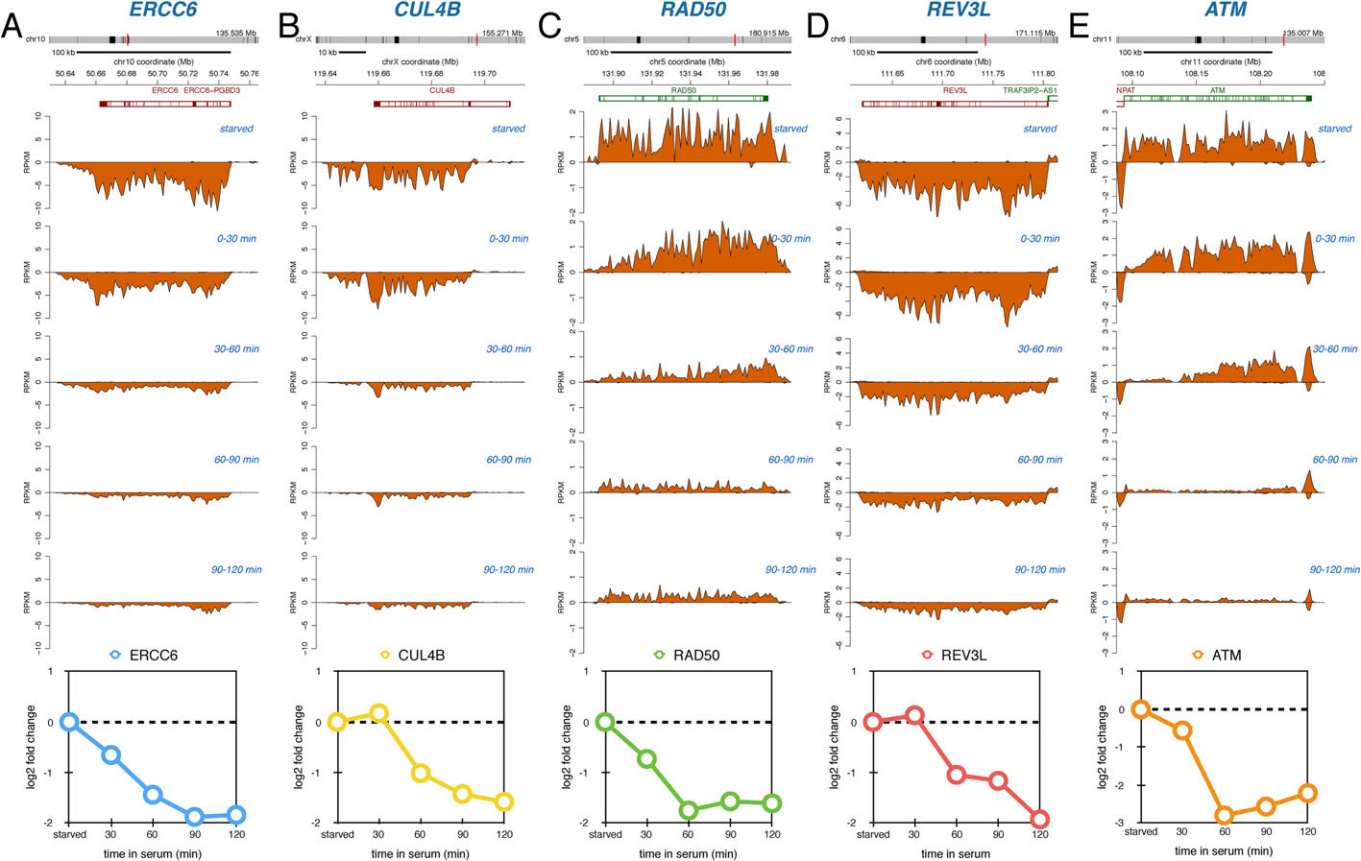
**DNA damage response genes are downregulated in response to serum.** Nascent RNA sequencing reads for *ERCC6* (A), *CUL4B* (B), *RAD50* (C), *REV3L* (D) and *ATM* (E) during starved conditions and during different periods following serum addition. Data representation as in Fig. 2.

As observed in the GSEA analysis, transcription of a number of genes involved in DNA damage repair and signaling were rapidly suppressed following serum stimulation (Fig. 7). Both *ERCC6*, which encodes the CSB protein involved in transcription-coupled repair, and *CUL4B*, which encodes an ubiquitin ligase implicated in DNA repair, were rapidly repressed. *RAD50,* which encodes a protein in the DNA double-strand break processing complex MRN, and *REV3L,* which encodes the catalytic subunit of translesion DNA synthesis polymerase zeta, were also suppressed during the first two hours following serum stimulation. Finally, transcription of *ATM,* which encodes a DNA damage response kinase, was suppressed after serum addition. The functional implications of the downregulation of these DNA damage response genes during the serum response are not known.

### AP-1, circadian clock, miRNAs and p53 response genes are affected by serum

The AP-1 transcription factor components FOS and JUN are known to be rapidly induced following serum stimulation (Herschman, 1991). Bru-seq revealed that six out of seven of these genes were rapidly induced after serum addition followed by a reset of nascent transcription, with the exception of *JUNB,* which was induced and sustained throughout the 2 h period after serum addition (Fig. S4). The mechanism of this burst of transcription followed by a reset may stem from the release of RNA polymerases from a promoter-proximal pause site, as is known for *FOS* (Liu et al., 2015; Plet et al., 1995), or the activation of inhibitory factors suppressing subsequent rounds of transcription, like *FOSL1* (Hoffmann et al., 2005). Serum stimulation inhibited transcription of a number of p53-response genes either immediately or after a short delay (Fig. S5). Serum starvation has been shown to increase expression of p53, with subsequent decrease after re-addition of serum (Hasan et al., 1999). Circadian clock genes also responded to serum stimulation, with some genes showing sustained induction and others showing a transient induction (Fig. S6). Lastly, Bru-seq analysis allows for the identification of miRNA transcription units, and we observed that serum stimulation affected nascent transcription of a number of miRNAs such as miR21 (Fig. S7).

### Transcription accelerates towards the 3′ end of genes

**Fig. 8.**
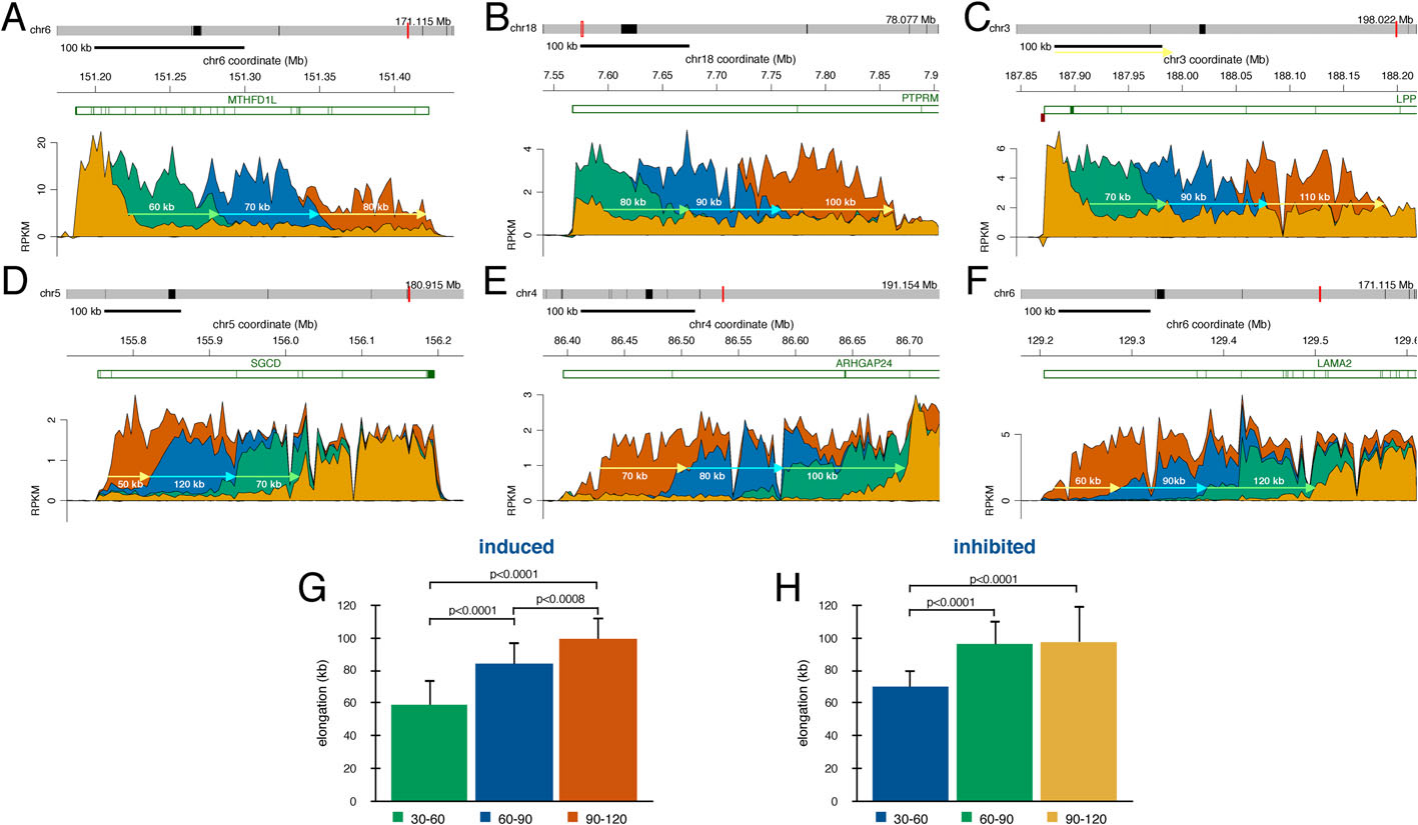
**Transcription accelerates during elongation.** Nascent RNA sequencing reads for large induced genes (A-C) during the different labeling periods (0-30 min yellow, 30-60 min green, 60-90 min blue, 90-120 min orange). Nascent RNA sequencing reads for large inhibited genes (D-E) during the different labeling periods (0-30 min orange, 30-60 min blue, 60-90 min green, 90-120 min yellow). Estimated distances of each transcription wave are indicated over or under each arrow. The mean of the estimated elongation rates of 21 genes with standard deviations are displayed for (G) induced and (H) inhibited genes for the different labeling periods. The *P*-values are indicated above each comparison.

Extremely long genes are estimated to take several hours for the completion of transcription, however, it has been suggested that elongation rates accelerate as RNAPII travels across the gene body and this could act to reduce transcriptional delays (Danko et al., 2013; Jonkers et al., 2014). We previously used BruDRB-seq to assess transcription elongation rates in the beginning of large genes in the same fibroblast cell line used in this study (Veloso et al., 2014). For very large genes (>200 kb) we could estimate the elongation rates during each 30 min time interval following serum stimulation by measuring the change in the location of the front of the transcription wave. We found that the first 30 min Bru-labeling interval generated large variability in the distances travelled by RNAPII probably due to variability in transcription initiation timing. Therefore, we excluded the first time interval and instead examined the distances traveled by the transcription wave during the three later labeling periods. Transcription rates for each gene were calculated using the genomic distance between leading edges of transcription waves between two time intervals divided by the 30 min labeling time. The median distance covered by the transcriptional wave across 21 induced genes was 60 kb, 90 kb, and 100 kb during the 30-60, 60-90, and 90-120 min labeling periods, respectively. This translates into an average elongation rate of 1.97 kb/min, 2.79 kb/min, and 3.32 kb/min for the respective time intervals and supports the idea that RNAPII accelerates as it travels across large genes (Fig. 8A-C,G; Figs S8, S9). Long genes repressed by serum exhibited a retreating wave of transcription which was used to measure elongation rates in a similar way. In 21 repressed genes, RNAPII was estimated to travel a median distance of 70 kb, 90 kb, and 100 kb during the 30-60, 60-90, and 90-120 min labeling periods, respectively. The corresponding elongation rates were 2.33 kb/min, 3.19 kb/min, and 3.25 kb/min for the respective time intervals (Fig. 8D-F,H; Figs S10, S11). These findings suggest that transcription complexes accelerate as they traverse large genes, but that these increased elongation rates are not the result of gene induction caused by stimulation by serum but rather an inherent feature of transcription over long distances.

## DISCUSSION

During a global cellular response, cells undergo widespread expression changes in order to modulate their function to adapt to changes in their environment. The ability to successfully follow the temporal chain of events occurring during a particular cellular response is critical for understanding the complex regulatory networks which orchestrate these gene expression changes. Categorizing response genes which display similar expression patterns can assist in identifying common regulatory mechanisms. Regulation of transcription initiation is a key step in establishing precise temporal expression of response genes, however, transcription elongation and RNA processing events create time delays for the generation of full-length mature mRNAs, and these delays are especially apparent for long genes. Therefore, by capturing nascent transcriptome dynamics and focusing on transcription initiation events immediately following cellular stimulation, we can more accurately assess early regulatory patterns and better model the mechanisms responsible for these changes.

In this study we present the first genome-wide data set of nascent transcription changes during serum activation. Our characterization of the early serum response is a reflection of temporal patterns of productive initiation because our focus was on transcription occurring near TSSs. This classification is distinct from previous studies that used steady-state RNA or mRNA detection and therefore categorized response genes based on transcript completion rather than initiation of transcription (Amit et al., 2007; Iyer et al., 1999; Tullai et al., 2007). We identified 1417 induced and 636 repressed genes during the first two hours following serum addition, and these genes displayed diverse transcriptional patterns. We identified a set of genes that showed an immediate transcriptional response and genes that responded after a delay. We were also able to categorize genes based on the behavior of their transcriptional initiation over time, including whether the change in RNA initiation was sustained or transient. Genes that exhibit similar transcriptional patterns may be subject to the same mechanisms of transcriptional regulation. For example, at the level of transcription initiation, response timing might be related to enhancer status at the time of stimulation. Primed enhancers might regulate rapidly induced genes while *de novo* enhancer selection might result in a delayed response. Also related to initiation, different sets or combinations of transcription factors or repressors may be responsible for immediate responses compared to delayed responses. Alternatively, regulation at the level of promoter-proximal pausing and release may also regulate response timing. Whether a transcriptional response is transient or maintained may be a factor of whether negative feedback mechanisms act to detect and limit RNA production. While this study did not explore specific mechanisms of transcriptional control, it provides a foundation for future studies by establishing candidate gene sets that may be coordinately regulated.

By following nascent transcription genome-wide over the first 2 h after serum addition, we observed a number of very diverse gene activation responses. One set of genes was immediately induced and showed sustained enhanced transcription initiation throughout the time course (Fig. 2A,B, Fig. 5B). This behavior likely reflects genes that are activated by transcription factors that have sustained activity in the presence of serum. Another set of genes showed a transient induction pattern (Fig. 3A,B, Fig. 5C). It is possible the transient induction pattern observed is due to a serum-mediated release of RNAPII from promoter-proximal arrest sites (Adelman and Lis, 2012; Liu et al., 2015; Muse et al., 2007). Interestingly, following this release these genes did not re-fire even though the continued presence of serum should maintain activating signals, thus cells appear to have the ability to limit the expression of these genes to one ‘wave’ of transcription through the genes. Alternatively, transient activation of a transcription factor or serum-induced transcription of a small gene encoding a transcription suppressor could explain the transient nature of expression. Another common pattern of gene transcription after serum stimulation is a delayed transcriptional response (Fig. 4A,B, Fig. 5D). These genes are most likely dependent on the immediate induction of transcription factor genes where the time delay of the induction of the secondary response genes is proportional to the sizes of the initially induced transcription factor genes. Finally, a fairly abundant group of genes showed a ‘reversal’ pattern where these genes were initially repressed but then showed highly induced transcription initiation (Fig. 5E). Such a pattern could potentially be generated by a feedback loop mechanism where cells compensate for the initial repression by follow-up overexpression.

The biggest surprise of our study was that the dynamic transcription patterns we observed for genes induced by serum-stimulation was also observed in the opposite direction. This novel data set was made possible because the Bru-seq technique measures transcription activity and not steady-state RNA, so rapid gene repression can be assessed as readily as transcriptional activation. Similarly to gene induction, we observed a group of genes that showed sustained repression throughout the time course (Fig. 2C,D,Fig. 5G). Here the presence of serum may inactivate specific transcription factors and this inhibition is sustained resulting in the maintenance of reduced transcription initiation of target genes during the time course. The mechanisms responsible for the transient repression of transcription of a specific set of genes (Fig. 3C,D, Fig. 5H) is not fully clear to us, but it is possible that serum immediately activates promoter-proximal transient transcription arrest in this set of genes with the subsequent establishment of a new equilibrium leading to new transcription emerging throughout the gene after a delay. Alternatively, serum transiently activates a transcription repressor or transiently inactivates a transcription activator. The group of genes with delayed repression (Fig. 4C,D, Fig. 5I) may have binding sites for repressors encoded in genes that are activated in the primary wave of serum-induced transcription. Finally, as for the induced gene set, there was a subset of genes that showed a ‘reversal’ behavior. After a transient induction these genes were suppressed at the later time points (Fig. 5J) and it is possible that these genes were regulated by a homeostatic feedback mechanism.

We hypothesize that these different transcriptional patterns are important for establishing temporal expression patterns related to functional activity. We performed DAVID and GSEA analyses to explore pathway and gene set enrichment patterns during the early serum response. We found that pathways which were highly enriched during the first labeling period, in either the group of upregulated or downregulated genes, tended to be enriched during the later labeling periods as well. Many of these enriched pathways were signaling pathways, such as the MAPK pathway, which is known to be activated by growth factor stimulation (Assoian and Schwartz, 2001). Attenuation of MAPK signaling and downstream target gene expression has been shown to depend on nascent transcription following activation (Amit et al., 2007); therefore the enrichment of genes in these pathways may be associated with transcriptional changes related to turning off the signaling pathway. MAPK signaling activation results in transcriptional activation of AP-1 components *FOS* and *JUN* (Shaulian and Karin, 2002), which we were able to observe using Bru-seq. Induction of these genes is accompanied by the upregulation of alternate AP-1 components which may inhibit active AP-1 complexes, as has been shown for FOSL1 and JUNB (Hoffmann et al., 2005; Szabowski et al., 2000). The detection of genes induced or repressed within the same time frame may help to identify novel regulators. The transcriptional response timing for these regulators is likely influenced by many factors, including RNA and protein abundance; therefore simultaneous assessment of transcriptional and translational regulation may be necessary to connect functional activity to temporal gene expression regulation.

Interestingly, we observed a significant downregulation of p53 response and DNA damage response genes following serum stimulation. Protein levels of p53 are known to increase in serum-starved cells due to enhanced protein stability, but p53 protein levels return to baseline following addition of serum (Hasan et al., 1999; Shi et al., 2012). The decrease of p53 target genes following serum addition is therefore likely related to the expected lower protein levels of p53. Our Bru-seq data detected repression of the p53 target gene *CDKN1A,* encoding the G_1_ cell cycle arrest protein p21, which has also been observed to have increased levels in starved cells (Shi et al., 2012). Surprisingly, we observed decreased transcription of the DNA damage response signaling genes *ATM* and *RAD50* following serum stimulation. The functional implications related to the downregulation of these DNA damage response genes following serum activation will need to be explored in more detail.

Lastly, our data demonstrated that elongation accelerates as RNAPII travel across long genes, which is consistent with previous studies (Danko et al., 2013; Jonkers et al., 2014). Since average elongation rates were found to increase across both induced and repressed genes following serum stimulation, our results suggest that acceleration of transcription elongation is not due to serum activation but rather is an inherent characteristic of transcription elongation. The mechanism responsible for this accelerated elongation may be related to a gradual shift in the phosphorylation pattern of the c-terminal domain (CTD) of RNAPII as it traverses genes (Jonkers and Lis, 2015).

Taken together, this study provides a novel view of the dynamic transcriptome as cells adjust to a new environmental condition after serum addition. Bru-seq revealed both known and novel changes in gene transcription during the early serum response. The assessment of global transcriptional output provides clues as to how regulation of transcription is accomplished after cellular stimulation. We believe that nascent sequencing techniques, such as Bru-seq, will be critical to assess and untangle the mechanisms behind temporal and dynamic expression changes for a wide range of cellular responses.

## MATERIALS AND METHODS

### Cell culture and serum stimulation

HF1, hTERT immortalized foreskin-derived human fibroblasts (Paulsen et al., 2014, 2013), were grown in MEM supplemented with 10% FBS, L-glutamine, vitamin mix, and antibiotics. These cells are routinely tested for mycoplasma contaminations. Starved cells were grown in the same media minus FBS for 48 h. For serum stimulation experiments, FBS was added to the media of starved cells (final concentration 10%). Serum addition was followed by 30 min bromouridine labeling, either immediately or after a 30, 60, or 90 min incubation period. Bru-labeling was performed in serum-starved cells and at different time-intervals following serum addition as illustrated in Fig. 1.

### Bru-seq analysis

Bru-seq was performed as previously described (Paulsen et al., 2014, 2013). Briefly, bromouridine (Bru) (Aldrich) was added to the media of starved or serum stimulated cells to a final concentration of 2 mM and incubated at 37°C for 30 min. Total RNA was isolated using TRIzol reagent (Invitrogen), and Bru-labeled RNA was isolated by incubation of the isolated total RNA with anti-BrdU antibodies (BD Biosciences) conjugated to magnetic Dynabeads (Invitrogen) under gentle agitation at room temperature for 1 h. cDNA libraries were prepared from the isolated Bru-labeled RNA using the Illumina TruSeq library kit and sequenced using Illumina HiSeq sequencers at the University of Michigan DNA Sequencing Core. The sequencing and read mapping were carried out as previously described (Paulsen et al., 2014, 2013).

### Gene expression and serum response analysis

RPKM (reads per kilobase per million mapped reads) values were calculated for individual genes over 300 bp for starved cells and for each serum stimulated sample. For genes under 30 kb, RPKM was calculated using read counts from the entire gene. For genes 30 kb and over, an RPKM value was calculated using read counts from the first 30 kb downstream of the TSS. Genes were classified as expressed if they had an RPKM value greater than 0.5 in starved cells or in at least one serum stimulated sample.

To identify response genes, an inter-sample comparison analysis was done to obtain RPKM fold-change values for each gene in a given serum stimulated sample compared to the starved sample (Paulsen et al., 2014, 2013). Genes with a greater than twofold change in any serum stimulated sample compared to starved cells were categorized as serum-response genes. Genes with greater than a twofold increase were classified as induced and genes with greater than a twofold decrease in RPKM values were classified as repressed. Repressed genes were required to be expressed (RPKM value>0.5) during the starved condition.

For the generation of the heat maps, induced and repressed response genes were clustered based on when the first 30 kb of the gene reached a greater than twofold change compared to starved cells. Genes were also clustered based on whether the expression change was sustained or transient. Sustained response genes maintained a greater than twofold change in the labeling periods following the initial response. Transient response genes had expression levels that returned to starved levels (less than twofold change) during the labeling periods following the initial response.

### Gene enrichment analysis

We used DAVID Bioinformatics Resource 6.7 (Huang et al., 2009a,b) to perform gene set enrichment analysis on the serum-responsive genes. The background gene set used for the analysis contained all genes that were expressed above 0.5 RPKM in the tested cells. We performed a functional annotation analysis using induced and repressed response gene sets from each labeling period. Here we present enriched pathways with a *P*-value <0.05. We also performed gene set enrichment analysis (GSEA) (Subramanian et al., 2005). All genes expressed >0.5 RPKM were rank-ordered according to log2-fold changes compared to the gene expression in serum-starved cells.

### Elongation analysis

Images displaying read distributions across genes longer than 200 kb were visually assessed to estimate distances travelled by RNAPII in the 30-60, 60-90, and 90-120 min labeling periods according to the changing positions of the transcriptional wave. Twenty-one genes were analyzed for groups of induced and repressed genes, and mean, standard deviation, and median values were recorded for the different labeling periods. These values were used to calculate an average elongation rate for induced and repressed genes during each labeling period.
